# Plasmon-induced transparency sensor for detection of minuscule refractive index changes in ultra-low index materials

**DOI:** 10.1038/s41598-021-01246-x

**Published:** 2021-11-04

**Authors:** Shahriar Farhadi, Mehdi Miri, Ali Farmani

**Affiliations:** 1 School of Electrical and Computer Engineering, Shiraz University, Shiraz, Iran; 2 School of Electrical and Computer Engineering, Lorestan University, Khoramabad, Iran

**Keywords:** Biophysics, Optics and photonics

## Abstract

Detection of low-index materials such as aerogels and also detection of refractive index variations in these materials is still a challenging task. Here, a high figure of merit (FOM) sensor based on plasmon-induced transparency (PIT) is proposed for the detection of aerogel refractive index changes. In the proposed PIT sensor, the transparency window in an opaque region arises from the coupling between surface plasmon polariton (SPP) mode and planar waveguide mode. By comprising sub-wavelength grating (SWG) in the planar waveguide region, the maximum of the electric field of waveguide occurs in a low index media. This facilitates detection of the aerogels when they are used as the low index material (sensing material). Application of the subwavelength grating waveguide also improves the sensitivity of the sensor by a factor of six compared to a conventional structure with a homogenous waveguide. The proposed structure has a quality factor of *Q* ≥ 1800, and a reflection of 86%, and can detect the refractive index changes as low as Δ*n* = 0.002 (around *n* = 1.0). The lineshape, *Q*-factor, and resonant wavelength of the transparency spectrum can be controlled by tailoring the structural parameters. Our work also has potential application in switching, filtering, and spectral shaping.

## Introduction

Induced transparency is a phenomenon that originates from destructive interference between a narrow and a wide spectrum resonance. As a result, a sharp transmission peak appears in broad absorption band^1,2^. The abrupt phase dispersion in the induced transparency is beneficial in a variety of applications such as slow-light, nonlinear optics, and sensing^3–5^. Induced transparency in optical systems is usually realized in form of electromagnetic induced transparency (EIT)^6^. EIT is a quantum coherent process that was first observed in three-level atomic systems^6^. In optical systems, the EIT results from the interference between a narrowband (dark) mode and a wideband (bright) mode. Plasmon-induced transparency (PIT) is an analog of the EIT phenomenon in metamaterial structures. In PIT, broad low-quality resonance is provided by surface plasmon polariton (SPP) mode at the metal–dielectric interface, and a dielectric waveguide mode is responsible for realizing a narrow high-quality resonance^7,8^. To date, various materials and structures have been investigated for the realization of EIT and PIT, such as ultra-cold atomic gas^9^, metamaterials^10^, and microcavities^11^. In PIT systems, SPP mode is directly excited by incident light and called bright mode. In contrast, the dark mode cannot couple to the incident field and is excited through the evanescent field of the SPP mode. As mentioned, the destructive interference between bright and dark modes induces a sharp transmission window in the wide absorption band^12^.

Plasmonic metamaterial^13,14^ and metasurface^15–21^ structures have been widely studied for sensing applications. The sensitivity and resolution of conventional plasmonic sensors are limited by inherent optical loss of the metals which results in wideband SPP resonance. To address this drawback, a variety of solutions have been proposed to obtain narrow linewidth resonances. Among these, the realization of PIT has drawn considerable attention in sensing applications because of its spectral characteristics^22,23^. Planar waveguide-coupled SPP structures have been widely used as promising candidates for realizing PIT^24,25^. However, in these structures, the field of the narrow spectrum mode (dark mode) is mainly confined in a dielectric media. Therefore, in applications where the sensing medium is air or an ultra-low index material such as aerogels the sensitivity of these structures is relatively low.

It should be noted that the inherent loss of the metallic material used for the realization of the SPP still exists in the PIT structure which degrades the overall performance of the PIT in terms of sensitivity and *Q*-factor. Although this limitation cannot be completely mitigated, the *Q*-factor and sensitivity of the PIT sensors can be improved in the design process as it is done in the following.

Aerogels, as an ultralight-materials, have received great attention owing to their unique features including low density, high internal surface area, and low thermal conductivity^26^. Aerogels are prepared by removing the solvent in the sol–gel by exploiting specific drying techniques to conserve the porous network^27^. Aerogels are widely employed in Cherenkov detectors^28^, thermal insulation^29^, air cleaning^3^, catalysis^30^, and sensors^31^. Also, because of their low refractive index of 1.007–1.240^32^, these materials have been studied for application in optical devices. For example, Dongheok Shin et al. reported an aerogel-based macro-scale transformation-optics wave bender and Lunburge lens in visible wavelength range^33^. Limin Tong and his colleagues employed the silica aerogel as a substrate to assemble low-loss nanoscale optical waveguides^34^. In 2018, Yeonhong Kim et al. utilized the silica aerogel as low index material to enhance the sensitivity of plasmonic sensors^35^.

The application of aerogels as the sensing material of the PIT sensors could expand their range of application. However, PIT sensors with air or aerogel sensing material have not been realized yet. This limitation, as mentioned above, arises from the structures of the previously studied PIT sensors in which the dark mode is confined in a high-index media. Here, a high figure of merit PIT sensor in silicon-on-insulator (SOI) platform is proposed and evaluated numerically in which the maximum of the dark mode electric field occurs in an ultra-low refractive index media. In the proposed sensors instead of a planar waveguide, the dark mode is produced in a subwavelength grating (SWG) waveguide. It is observed that by replacing the homogenous planar waveguide with an SWG waveguide, the sensitivity can be improved by a factor of 6 compared to the previous PIT sensors. We have also examined the effects of geometrical parameters on the *Q*-factor, lineshape, and resonant wavelength of the PIT effect.

## Results

**Figure 1:**
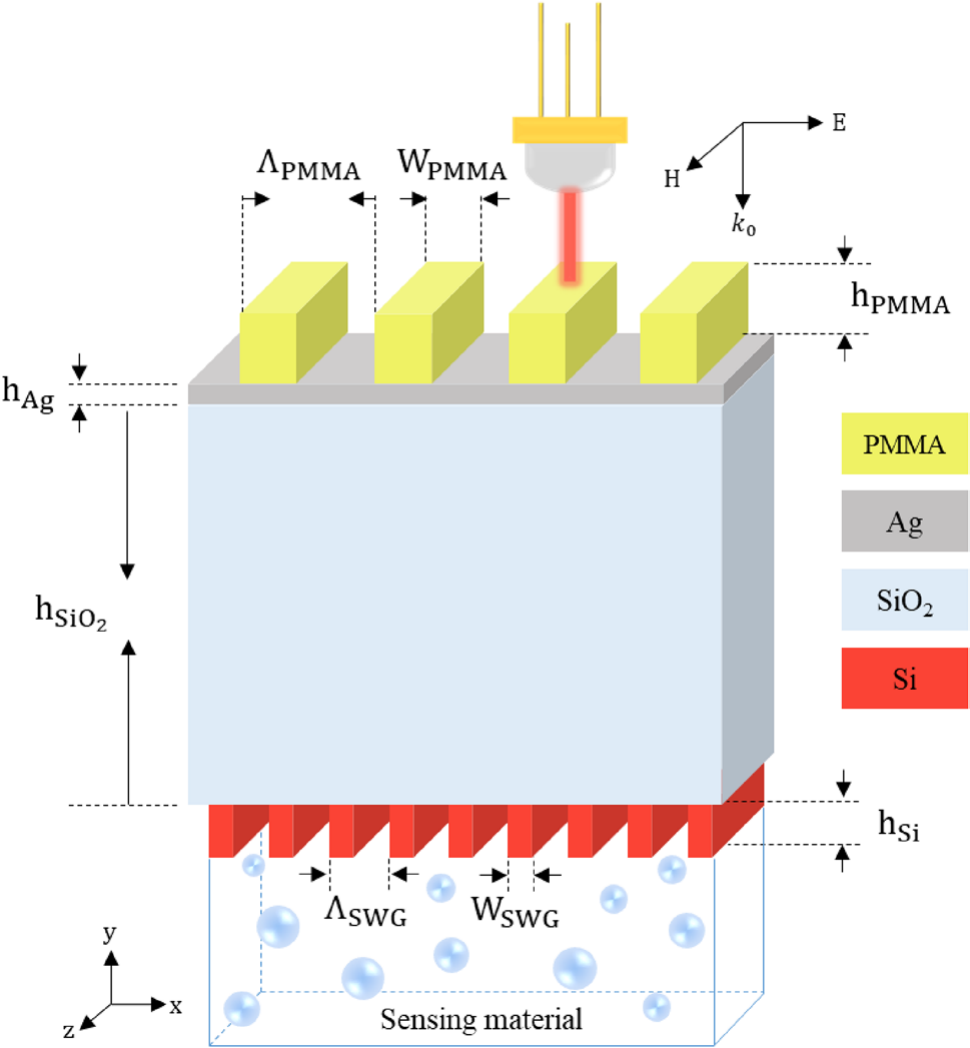
Schematic configuration of the proposed plasmonic system.

The schematic configuration of the proposed PIT sensor is depicted in Fig. 1. It consists of a PMMA grating and a thin Ag film coated on an SOI wafer. The interface of Ag film and SiO_2_ supports the SPP mode, and the SWG waveguide is made up of a stack of SiO_2_, Si grating and, sensing material. The prism coupling method has been widely used in Kretschmann configuration^36^ to excite SPP, but it has a large dimension and requires precise incident angle adjustment which hinders its application. To address this issue, we adopted a PMMA grating to compensate wavevector mismatch between normal incident light and the SPP mode. Under the incident of transverse magnetic (TM: with magnetic field normal to the plane of the incident) polarized light, SPP mode is excited. To characterize the designed plasmonic structure, two-dimensional finite difference-time-domain (2D FDTD) simulations have been utilized. The PMMA grating pitch (Λ_**PMMA**_) and Ag thickness (h_Ag_) were chosen 700 nm and 20 nm, respectively, to achieve a minimum of reflection.

The SOI platform is selected in our design because of the strong field confinement of this platform and also its compatibility with highly mature and cost-effective CMOS technology^37,38^. As mentioned, SWG waveguide is used in our design for the formation of the narrowband mode instead of the typical planar waveguide. SWGs are periodic structures with grating pitch (Λ_SWG_) much smaller than the operating wavelength (Λ_SWG_ << λ) and surpass the diffraction limit^39^. SWG structure can be fabricated by high precision electron-beam lithography (EBL) technology. A suggested fabrication process flow for the realization of the proposed structure is provided at the end of this manuscript in the Methods section.

**Figure 2:**
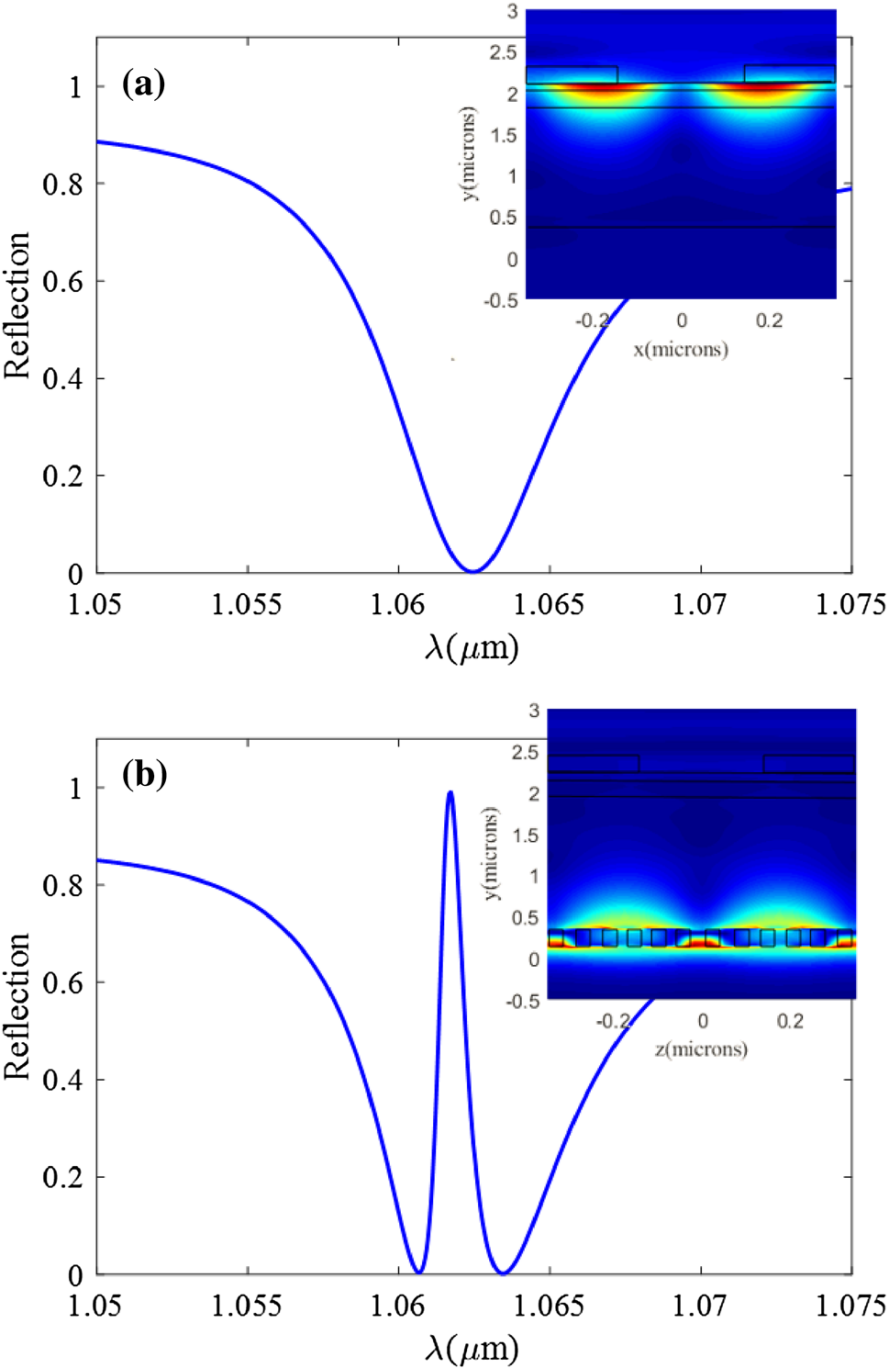
Reflection spectra and electric field distribution. (**a**) The reflection spectrum of the structure without Si SWG. The inset shows the electric field distribution at the wavelength of λ = 1063 nm. (**b**) The reflection spectrum of structure with Si SWG. The inset depicts the electric field distribution at λ = 1061.6 nm. The figures are calculated for the geometrical parameters of Λ_PMMA_ = 700 nm, W_PMMA_ = 320 nm, h_PMMA_ = 250 nm, h_Ag_ = 20 nm, ${\text{h}}_{{{\text{SiO}}_{2} }}$ = 1735 nm, Λ_SWG_ = 170 nm, W_SWG_ = 88 nm, h_Si_ = 220 nm, and the sensing material of air.

In our simulations, the refractive indices of Si and SiO_**2**_ layers are assumed to be 3.476 and 1.445, respectively, and refractive indices of Ag and PMMA are also adopted from Ref.^40^ and^41^, respectively. Reflection spectra of the structure in absence of the silicon SWG are shown in Fig. 2a. The broad reflection dip in this figure represents the low-quality resonance or the dark mode which originates from the coupling between the incident light and the SPP mode. The field profile of the structure in the inset of Fig. 2a shows that without the silicon SWG layer, the electric field is confined at the Ag/SiO_2_ interface (in SPP mode).

When the silicon SWG waveguide is added to the structure as in Fig. 1, the guided mode of this waveguide can be coupled to SPP mode through the evanescent field. The coupling strength between dark and bright modes can be increased by the proper design of the structural parameters. As mentioned in the Introduction section, the coupling and interference of the SPP and SWG waveguide modes induce a sharp peak in the absorption band of the SPP structure. This sharp peak in the reflection which is usually called the transparency window can be seen in Fig. 2b. This figure displays the reflection spectra of the structure in presence of the silicon SWG (structure of Fig. 1). A comparison between Fig. 2a, b, shows that a transparency window (around  λ ~ 1061.6 nm) appears in the absorption spectra as a result of the coupling of SPP and SWG modes. According to the field profile in the inset of Fig. 2b, at the transparency wavelength of *λ* = 1061.6 nm, the electromagnetic field (and power) is mainly confined in the silicon SWG region. The SPP mode is almost canceled out due to destructive interference between this mode and the SWG waveguide mode. This can be seen in Fig. 2b as the insignificant field strength at the Ag/SiO_2_ interface.

To quantify the sensing performance of a sensor, many evaluation criteria such as quality factor (*Q*), sensitivity (*S*), and figure of merit (FOM) can be utilized. The quality factor of the transparency window is defined as follows:

1 $$Q = \frac{{{\uplambda }_{0} }}{{{\text{FWHM}}}}$$

where λ_0_ and FWHM are the wavelength and full width at half the maximum of the transparency peak, respectively. FWHM is a crucial parameter because the resolution of the sensor is highly dependent on FWHM. The lower FWHM, the higher resolution is.

Sensitivity is the most important characteristic of a sensor, and it defines the sensing accuracy of the device. Sensitivity is evaluated by monitoring the shift of reflection peak caused by the change in the sensing material's refractive index. Sensitivity by intensity can be expressed as:

2 $$S = \frac{{{\Delta }R}}{{{\Delta }n}}$$

where ΔR is the change in reflectivity at a fixed incident angle and Δn is the refractive index change. Another critical parameter of a sensor device is the FOM and is calculated from the following formula:

3 $$FOM = \frac{{\text{S}}}{{{\text{FWHM}}}}$$

The structural parameters in Fig. 2 were chosen roughly to only demonstrate the PIT. To achieve better sensing performance from our structure, different geometrical parameters of the structure should be modified. Starting from the structure in absence of the silicon SWG, we first find the optimum geometrical parameters of the Ag and PMMA layers. Then we add the silicon SWG and find optimum SiO_2_ thickness. The geometrical parameters of the SWG will be selected at the last step.

**Figure 3:**
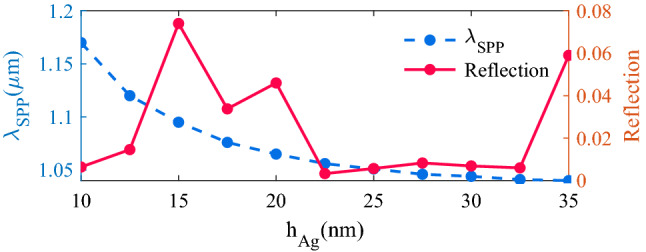
Effect of Ag thickness on the reflection and the resonance frequency of surface plasmon polariton (λ_SPP_).

In the first step, we set SiO_2_ and PMMA parameters to ${\text{h}}_{{{\text{SiO}}_{2} }}$ = 1500 nm, Λ_PMMA_ = 700 nm, W_PMMA_ = 320 nm, and h_PMMA_ = 250 nm. Then we calculated the reflection for different thicknesses of the Ag layer ranging from 10 to 35 nm. To obtain the highest detection accuracy, we should minimize the reflection in the absorption band as much as possible. So that, higher incident light energy transfers to SPP mode. This will result in stronger coupling and interference between SPP and SWG waveguide modes and a stronger transparency peak. As depicted in Fig. 3, with increasing the h_Ag_, the resonance frequency of surface plasmon polariton (λ_SPP_) is blue shifted. A thicker Ag layer increases the absorption loss of the structure and limits the sensitivity by increasing the FWHM. Also, the fabrication of a very thin Ag layer with high quality could be challenging. As can be seen in Fig. 3, for all values of h_Ag_, in the 10–35 nm range the reflection is lower than 0.1 and we set h_Ag_ to 20 nm to reach a reasonably low reflection and low optical loss.

**Figure 4:**
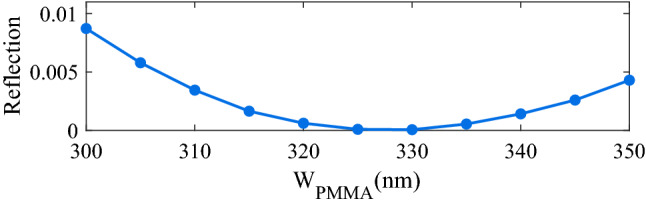
Effect of W_PMMA_ on the reflection for Ag = 20 nm, ${\text{h}}_{{{\text{SiO}}_{2} }}$ = 1500 nm, Λ_PMMA_ = 700 nm, and h_PMMA_ = 250 nm (calculated at  λ = 1063 nm).

In the next step, we swept W_PMMA_ from 300 to 350 nm to minimize the reflection. As we can see in Fig. 4, there is a minimum reflection of about 6.8 × 10^–5^ for W_PMMA_ = 330 nm. This nearly zero reflection means that the maximum power of the source is transferred to SPP mode.

**Figure 5:**
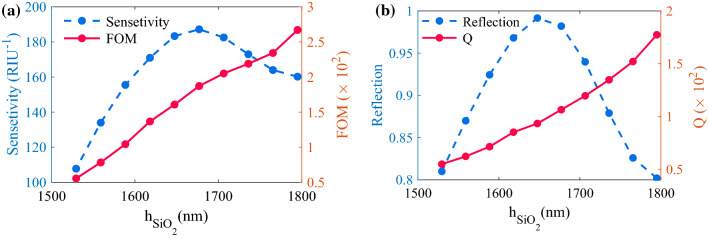
Effect of ${\text{h}}_{{{\text{SiO}}_{2} }}$ on (**a**) Sensitivity, and d FOM, (**b**) Reflection and Q.

Now, we analyzed the effect of SiO_2_ thickness on the sensitivity, FOM, and reflection peak of the structure to find the optimum h_SiO2_ value. To start, we set an initial value for SWG parameters as h_Si_ = 220 nm, Λ_SWG_ = 170 nm, and W_SWG_ = 88 nm. The his = 220 nm is a standard value for SOI wafers and other values are selected based on the condition that SWG supports a propagating mode in the abruption band of the structure (around *l* = 1063 nm). The changes in the FOM and sensitivity and also changes in *Q*-factor and reflection values (at  λ = 1063 nm) for 1530 nm ≤ ${\text{h}}_{{{\text{SiO}}_{2} }}$ ≤ 1800 nm are shown in Fig. 5a, b, respectively.

**Figure 6:**
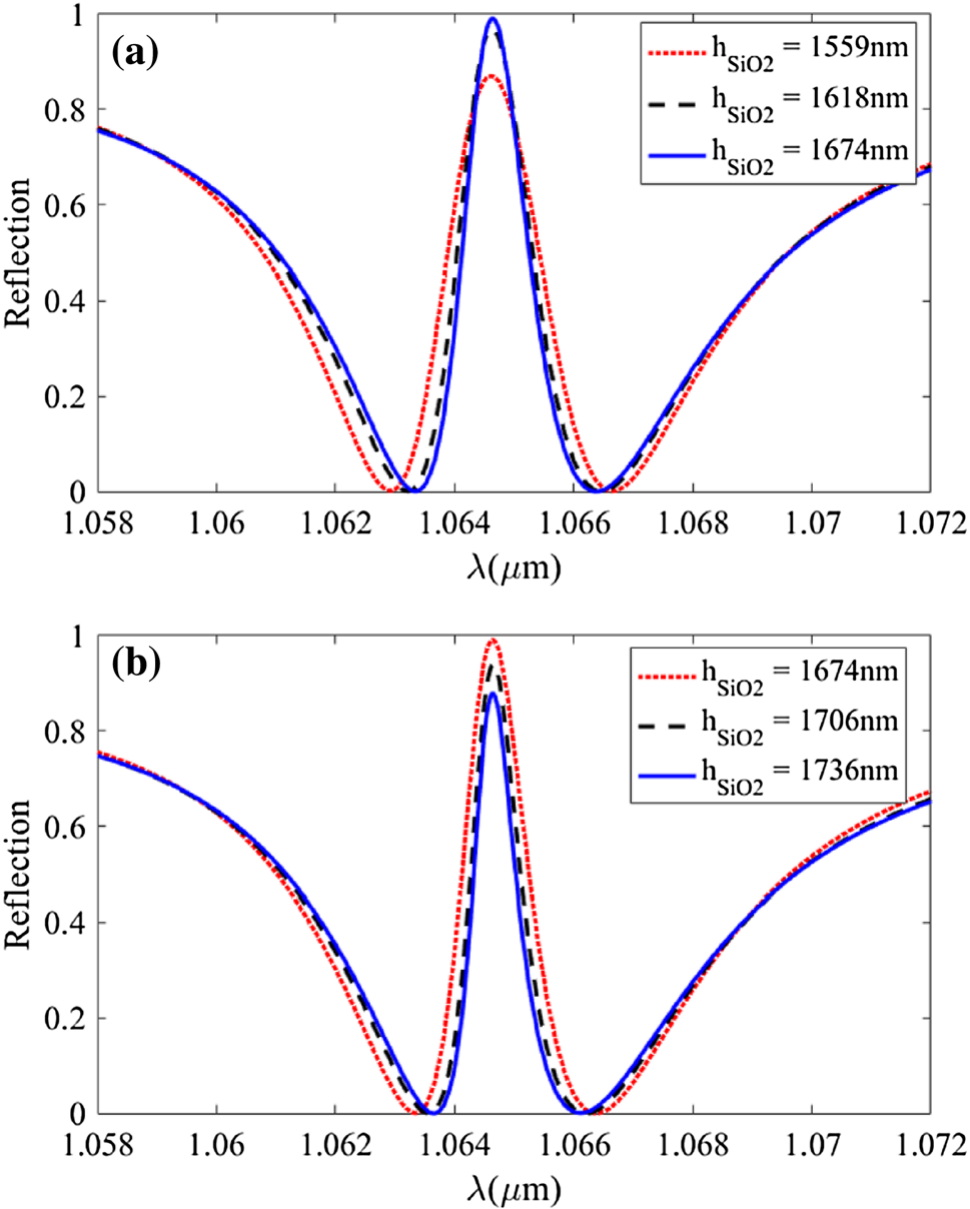
(**a**) and (**b**): Reflection spectra of the structure for different ${\text{h}}_{{{\text{SiO}}_{2} }}$ values.

As depicted in Fig. 5, by increasing the ${\text{h}}_{{{\text{SiO}}_{2} }}$ from 1530 nm, both the reflection and sensitivity increase and reach their peaks at ${\text{h}}_{{{\text{SiO}}_{2} }} \approx$ 1650 nm, and after that, they decline. Meanwhile, the quality factor and FOM follow a rising trend as the ${\text{h}}_{{{\text{SiO}}_{2} }}$ increases. The reflection of structure off Fig. 1 with h_Ag_ = 20 nm, Λ_PMMA_ = 700 nm, W_PMMA_ = 330 nm, h_PMMA_ = 250 nm, h_Si_ = 220 nm, Λ_SWG_ = 170 nm, and W_SWG_ = 88 nm is plotted in Fig. 6 for different h_SiO2_ values. As can be seen, the thickness of the SiO_2_ layer does not influence the transparency wavelength.

Based on the result of Figs. 5 and 6, we chose ${\text{h}}_{{{\text{SiO}}_{2} }}$ = 1800 nm so that reach the largest *Q*-factor and FOM while keeping the reflection and sensitivity at acceptable values.

**Figure 7:**
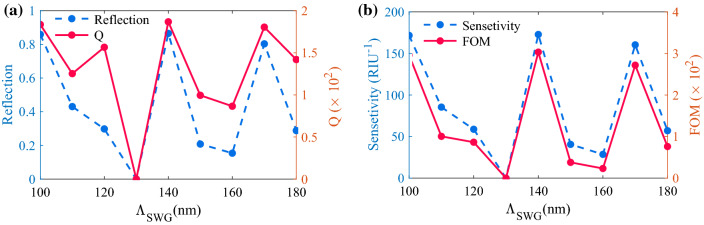
Effect of Λ_SWG_ on (**a**) reflection and Q, (**b**) Sensitivity, and d FOM.

In the last design step, to find the best geometrical parameters for SWG waveguide, we set h_SWG_ = 220 nm, which is a standard value of SOI wafers, and then vary Λ_SWG_. Due to the limitations of the lithography techniques, we set the minimum h_SWG_ to 100 nm. For each period, we adjust the W_SWG_ in a way to put the PIT (transparency) peak at λ = 1064.6 nm (mid wavelength of the absorption band) and then calculate the quality factor, sensitivity, FOM, and reflection peak. As demonstrated in Fig. 7 for Λ_SWG_ = 140 nm, we can obtain the highest values for the mentioned parameters. For Λ_SWG_ values larger than 190 nm, the grating is not working in the subwavelength regime. Therefore, we chose Λ_SWG_ = 140 nm and W_SWG_ = 73 nm as the final parameters for SWG which result in reflection of R = 0.865, *Q* = 1870, the sensitivity of *S* = 173 RIU^-1^, and FOM = 3.3 × 10^2^.

**Figure 8:**
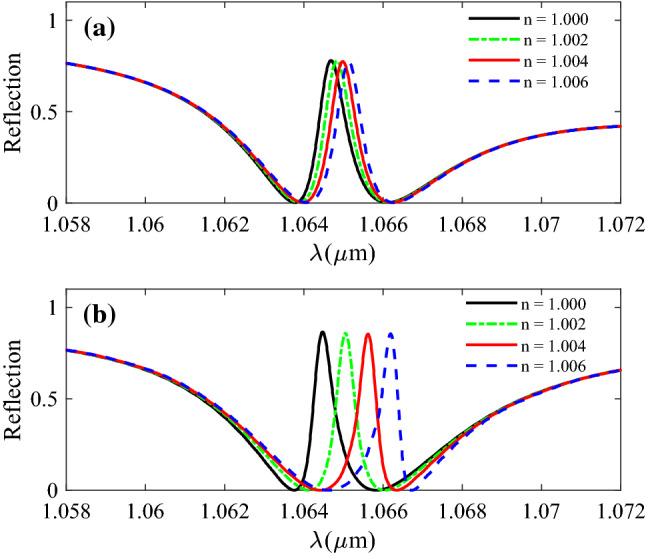
Reflection spectra of the plasmonic system for refractive index change with the step of 0.005 when (**a**) a homogenous silicon layer is used (h_Si_ = 110 nm). (**b**) Silicon SWG is used.

To evaluate the sensing performance of the final design with; h_Ag_ = 20 nm, Λ_PMMA_ = 700 nm, W_PMMA_ = 330 nm, h_PMMA_ = 250 nm, h_SiO2_ = 1800 nm, h_Si_ = 220 nm, Λ_SWG_ = 140 nm, and W_SWG_ = 73 nm, we have increased the refractive index of the sensing material by the step of 0.002 (D*n* = 0.002) and calculated the reflection. Figure 8a shows the reflection spectra of the sensor with a homogeneous waveguide mode as the narrowband mode instead of the SWG. As we can see, there is a very small red shift in resonance peaks caused by the increase in the refractive index of sensing material. On the other hand, when the silicon SWG waveguide is used to form the narrowband mode, a large shift in resonance peaks is observed in Fig. 8b. According to our calculations, the application of the SWG instead of a homogeneous waveguide has increased the sensitivity by a factor of six. The enhancement of the sensitivity in the proposed structure is because the effective surface area in contact with sensing material is remarkably enhanced by the application of the SWG.

**Figure 9:**
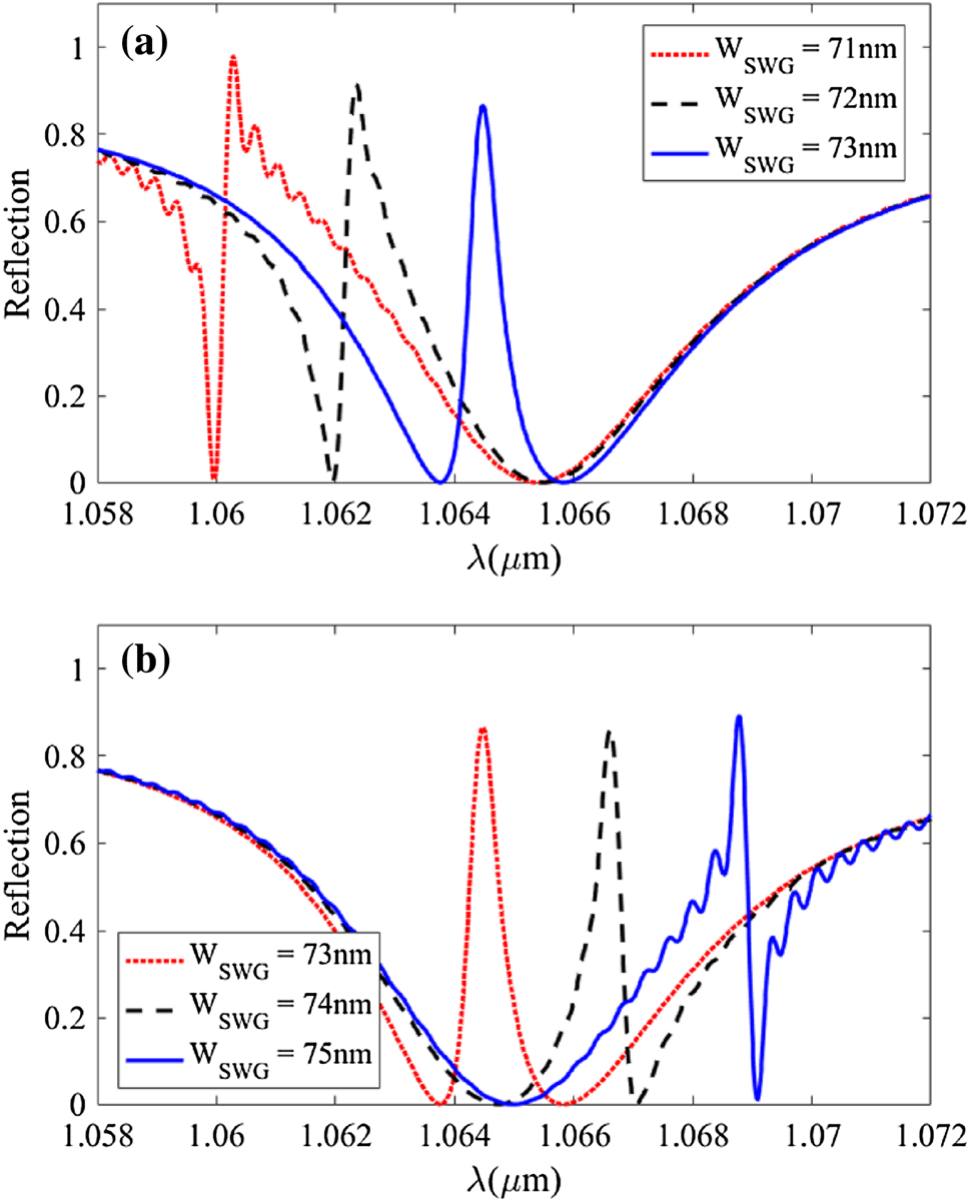
(**a**) and (**b**): Reflection spectra of PIT response corresponding to different subwavelength grating widths (W_SWG_).

Finally, we analyzed the effect of small variations in the SWG width (W_SWG_) on the reflection spectrum. These small variations which can be caused by the fabrication errors also occur in other geometrical parameters but, since the W_SWG_ variations have the most profound effect on device performance we have only studied this parameter. As can be seen in Fig. 9, the transparency peak is very sensitive to W_SWG_. Any small increment in W_SWG_ results in a drastic redshift in transparency peak and changes the PIT lineshape to Fano lineshape.

## Comparison

The performance of the proposed sensor is compared against some of the previous work in Table 1. Considered performance parameters include sensitivity, FOM, *Q*-factor, and minimum distinguishable refractive index change (Δ*n*_*d*_). FOM and Δn_d_ are usually considered as the most important sensor parameter. As reported in Table 1, our designed sensor shows better performance than the majority of counterparts.

**Table 1:** Comparison of the proposed sensor with some of the reported structure.

References	Sensitivity	Quality factor	FOM	Δn_d_
Ref ^42^	42.7	Not reported	3.84 × 10^4^	0.03
Ref ^43^	765	Not reported	3.326 × 10^4^	2 × 10^–3^
Ref ^44^	267.25	481.8	178.2	0.04
Ref ^45^	800	Not reported	61.55	0.01
Ref ^46^	7724.9	Not reported	92.3	0.01
Ref ^47^	497.8	12.88	480	0.2
This work	173	1870	3.3 × 10^2^	2 × 10^–3^

## Discussion

We have proposed and analyzed a modified planar PIT system based on the SOI platform for sensing small variations in the refractive index of aerogels. We have observed that by introducing the SWG waveguide instead of a homogeneous waveguide for the realization of the narrowband mode, the sensitivity of the PIT sensor can be increased at least by a factor of six. The quality factor of more than 1800 and FOM of over 3.3 × 10^2^, have also been achieved by the proposed structure. The proposed sensor is capable of detecting refractive index changes as low as 0.002 in low index materials such as aerogels. The presented structure has practical applications in sensing, filtering, switching, and spectral shaping.

## Methods

### Fabrication process

**Figure 10:**
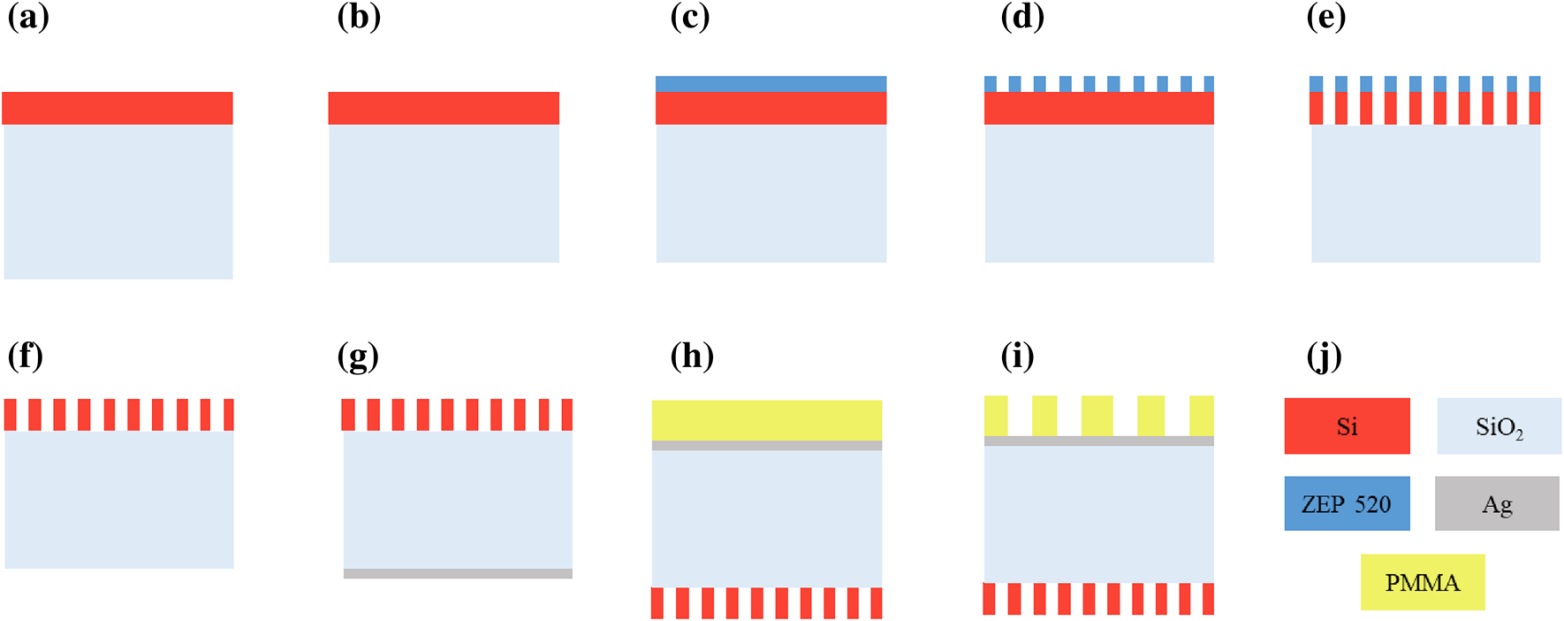
Suggested fabrication process flow for the proposed device.

Figure 10 depicts a suggested process flow for the fabrication of the proposed device. The device is implemented on a standard SOI wafer with 2 μm of silicon oxide (SiO_2_) and a silicon thickness of 220 nm (Fig. 10a). First, the SiO_2_ layer should be etched to the desired thickness by a chemical technique (Fig. 10b). Then, an electron-beam resist such as ZEP 520 should be spin-coated on the silicon layer (Fig. 10c). In the next step as in Fig. 10d high precision electron-beam lithography (EBL) technique will be utilized to form the SWG pattern. To etch through the silicon layer a selective etching technique such as reactive ion etching should be used and the residual resist will be removed (Fig. 10e, f, respectively). In the next step, a thin layer of Ag should be deposited on SiO_2_ through evaporation or sputtering process (Fig. 10g). Finally, the PMMA layer, a negative tone electron-beam resist, will cover the Ag layer and then (EBL) technique should be used to pattern the PMMA^48^ (Fig. 10h, i, respectively).

### Measurement

**Figure 11:**
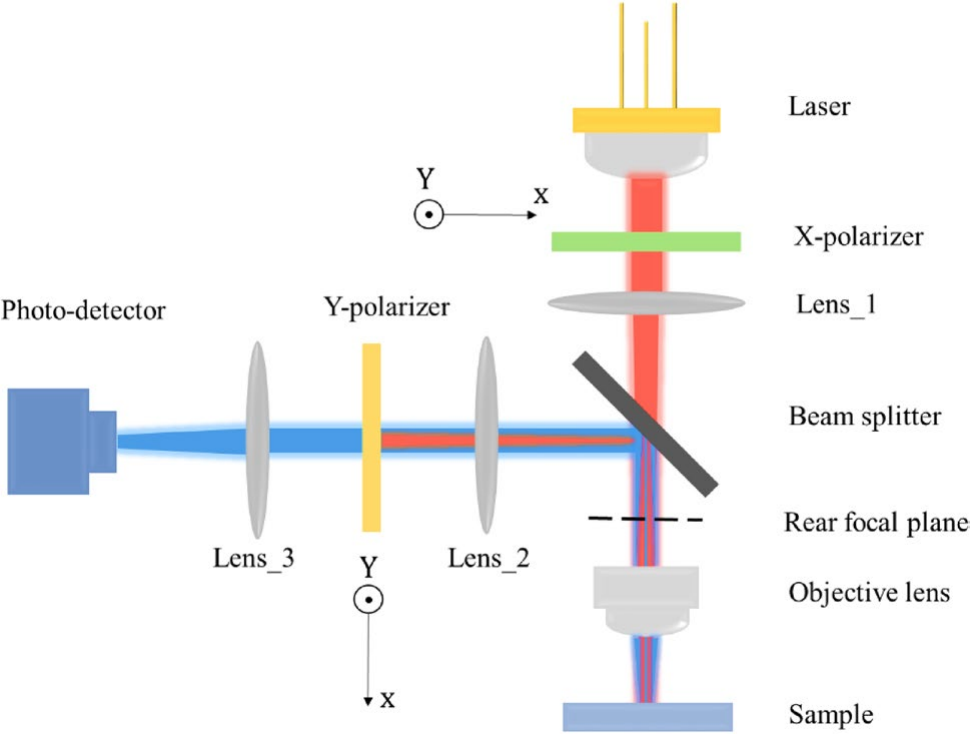
Schematic representation of the suggested experimental setup.

To characterize the sensing performance of the proposed device a measurement setup such as the one reported in Ref^49^ can be used. As illustrated in Fig. 11, the incident light (Red line) from a tunable laser passes through an X-polarizer and is focused by Lens_1 onto the rear focal plane. The objective lens focuses the incident light on the sample and collects the reflected light. The reflected light is sent through a relay 4f. system and magnified light is detected by a photodetector. Y-polarizer blocks X-polarized reflected light.

## Data Availability

The calculated results during the current study are available from the corresponding author on reasonable request.
